# Effect of ultrasound-pretreated starch on the formation, structure and digestibility of starch ternary complexes from lauric acid and β-lactoglobulin

**DOI:** 10.1016/j.ultsonch.2024.106990

**Published:** 2024-07-14

**Authors:** Bin Niu, Yingnan Qin, Xinhua Xie, Bobo Zhang, Lilin Cheng, Yizhe Yan

**Affiliations:** a College of Food Science and Technology, Henan Agricultural University, Zhengzhou 450000, PR China; b College of Food and Bioengineering, Zhengzhou University of Light Industry, Zhengzhou 450001, PR China; c National & Local Joint Engineering Research Center of Cereal-Based Foods (Henan), Zhengzhou, 450001, PR China

**Keywords:** Starch-LA-βLG complexes, Ultrasonic power density, Formation, Structure, Digestibility

## Abstract

Starch, lipids, and proteins are key macronutrients in starchy foods. Their interactions during processing can form starch-lipid-protein ternary complexes, significantly affecting food quality. Ultrasonic treatment, as a common processing method, is expected to regulate the quality of starchy foods by influencing the formation of ternary complexes. This study aimed to understand the effect of ultrasonic pretreatment on the formation of starch-lipid-protein ternary complexes using various types of starches. Wheat starch (WS), maize starch (MS), and potato starch (PS) were gelatinized and treated with ultrasound at various power densities (0–40 W/L) to form complexes with lauric acid (LA) and β-lactoglobulin (βLG), respectively. Ultrasound increased the amylose content of gelatinized WS, MS, and PS and shifted their chain length distribution towards the short chains. Results from Fourier transform infrared spectroscopy, laser confocal micro-Raman, X-ray diffraction, and differential scanning calorimetry showed that the largest amount of WS-LA-βLG complexes was formed at the ultrasonic power density of 10 W/L, and MS-LA-βLG and PS-LA-βLG complexes at 20 W/L. Additionally, ultrasound enhanced the content of resistant starch (RS) in the starch-LA-βLG complexes. The RS content increased from 14.12 % to 18.31 % for WS-LA-βLG, and from 19.18 % and 20.69 % to 27.60 % and 28.63 % for MS-LA-βLG and PS-LA-βLG complexes, respectively. This study presents an approach for facilitating the formation of ternary complexes, contributing to the development of low-GI functional foods.

## Highlights

• Ultrasonic treatment is used to facilitate formation of starch ternary complexes.
• Largest amount of WS-LA-βLG was formed at ultrasonic power density of 10 W/L.
• The optimal ultrasonic condition for MS-LA-βLG and PS-LA-βLG were 20 W/L.
• Ultrasonic treatment increased the amylose content of gelatinized starch.
• Ultrasound could improve the content of resistant starch in the starch complexes.

## 1 Introduction

Starch, a natural polysaccharide and composed of amylose and amylopectin [1], [2], is the primary source of carbohydrates and of great value in the human diet. Lipids and proteins are fundamental nutritional elements found in numerous food item, as well as being the main source of energy in the human diet. The interaction between starch and lipids can form starch-lipid binary complexes, which delay the starch gelatinization, increase the viscosity, and reduce the digestibility of starch [3], [4], [5]. Moreover, starch, lipids, and proteins can form ternary complexes, which further alter the structure and physicochemical properties of starch [5], [6]. Compared to starch-lipid complexes, starch-lipid-protein complexes show better long and short-range order, higher thermostability and viscosity [7], [8]. Negatively charged fatty acids has been considered as the bridge, which connect two macromolecules-amylose and protein in ternary complexes previously [9]. However, the recent research proved that the formation of the amylose-lauric acid-β-lactoglobulin ternary complex was primarily driven by hydrophobic interactions and van der Waals forces between amylose and lauric acid, and van der Waals forces and hydrogen bonds between amylose and β-lactoglobulin [10]. As a type of resistant starch (RS5), starch-lipid-protein complexes can effectively prevent rapid rise of blood glucose level, making them suitable for low glycemic index (GI) foods in diabetic diet and as fat substitutes in low-calorie foods. Therefore, there is a growing desire in increasing the content of starch-lipid-protein complex through appropriate processing method for healthier benefits [11]. For instance, increasing the pH [12] or the cooling rate [13] of the system could facilitate the formation of starch-lipid-protein ternary complexes. However, further research is needed to expand the use of physical methods to enhance ternary complex content.

In recent years, many studies have demonstrated that physical techniques, including microwave, ultrasound, ultra-high pressure, extrusion, and heat-moisture treatment, are extensively used in food processing to alter the functional properties of food [14]. As a non-thermal technology, ultrasound has been widely employed in food preservation, food testing, and food processing, especially for the production of modified starch [15].

Studies have shown that ultrasound affected starch particle size, granule morphology, chain interactions, crystalline structure, amylose content, thermodynamic properties, and resistant starch content [15], [16]. Higher ultrasonic frequencies increased amylose content and relative crystallinity of potato starch but decreased them in pea starch [17]. The particle size of maize, potato, and pea starches increased significantly after ultrasonic treatment [17]. For maize starch, the resistant starch content exhibited an initial increase followed by a subsequent decrease with the augmentation of ultrasonic power, indicating that ultrasound affected starch digestion characteristics [18]. Ultrasonic treatment could also enhance the formation of starch-lipid complexes [19], improving the thermal stability and diffraction intensity [20]. A study on pea starch and glycerol laurate (GM) complexes showed that long-term ultrasonic treatment and using ultrasonic treatment administered before the inclusion of GM produced more complexes [20]. The amount of complexes initially increased with rising ultrasound frequency but decreased with excessive treatment, which might be due to the disruption of the complexes or amylose [20], [21], [22]. The primary influence of ultrasonic treatment on starch was attributable to the high energy generated by ultrasonic cavitation accelerating the movement of starch granules, which caused different influence on granules and the crystalline/amorphous proportion of starch [21], [23]. Ultrasonic treatment could also disrupt the α-1,6 glycosidic linkage in starch, break the side chains in amylopectin, and increase amylose content, thereby generating more starch-lipid complexes [22].

In general, there were many studies focus on the influence of ultrasonic processing on physicochemical properties of starch or starch-lipid complexes. The extent of influence depended on treatment conditions, like ultrasonic power, time and so on [18], [20]. However, the impact of ultrasonic processing on starch-lipid-protein ternary complexes has been insufficiently explored in researches. Therefore, the impact of ultrasonic power density on the construction of ternary complexes with different types of starch was researched in this paper, followed by in-depth studies on the structural and digestive characteristics of the complexes. It is expected to regulate the formation and structure of starch-lipid-protein ternary complexes by adjusting ultrasonic conditions, thereby improving the quality and nutritive value of starch-based foods.

## 2 Materials and methods

### 2.1 Materials

Wheat starch (WS) was purchased from Shanghai Yuanye Biotechnology Co., LTD. (Shanghai, China). Maize starch (MS), potato starch (PS), lauric acid (LA, AR, 98 %), and β-lactoglobulin (βLG, BR, 95 %, from milk) were supplied by Shanghai Macklin Biochemical Technology Co., LTD. (Shanghai, China). Amylose/amylopectin kit (BC4265) was purchased from Solarbio Science & Technology Co., Ltd. (Beijing, China). D-Glucose assay kit (glucose oxidase/peroxide, GOPOD format) was supplied by Megazyme International Ireland Ltd. (Bray County, Wicklow, Ireland). Porcine pancreatic alpha-amylase (8 × USP, P7545), amyloglucosidase (260 U/mL, A7095), and isoamylase (1400 U/mL) were purchased from Sigma Chemical Co. (St. Louis, MO, USA). Other chemical reagents were all of analytical grade.

### 2.2 Ultrasonic pretreatment of starch

2 G of WS was diffused in 20 mL of deionized water to create starch paste using a thermostatic heated magnetic stirrer at 95 °C for 30 min. After gelatinization, the starch paste was treated by ultrasound (0, 10, 20, 30, and 40 W/L) at 50 °C for 30 min using an ultrasonic cleaner (KQ-800DM; kunshan ultrasonic instrument Co., Ltd., Kunshan, China) and named as WS-0, WS-10, WS-20, WS-30, and WS-40, respectively. According to the same procedure, starch pastes prepared from maize starch (MS) and potato starch (PS) was respectively named MS- and PS-ultrasonic power density. Those obtained starch pastes were freeze-dried, followed by milling into fine powder for the subsequent tests

### 2.3 Preparation of starch-LA-βLG complexes

After ultrasonic treatment of gelatinized starch, LA (100 mg) and βLG (200 mg) were incorporated to the above starch paste, and the mixture was heated by the thermostatic heated magnetic stirrer at 95 °C for 30 min [10], [13]. Thereafter, it was freeze-dried and ground to obtain starch-LA-βLG ternary complexes. The ternary complexes prepared from ultrasonic pre-treated WS paste, MS paste, and PS paste were named WS-LA-βLG-ultrasonic power density, MS-LA-βLG-ultrasonic power density, and PS-LA-βLG-ultrasonic power density, respectively.

### 2.4 Determination of apparent amylose content

An amylose/amylopectin assay kit (BC4265) of Solarbio was used to determine the amylose (AM) content of the ultrasonic pre-treated starch.

### 2.5 Size exclusion chromatography-refractive index (SEC-RI)

The ultrasonic pre-treated starch (5 mg) was dissolved in 0.9 mL of water in a boiling water bath for 15 min. Sodium azide solution (5 mL, 40 mg/mL), acetate buffer (0.1 mL, 0.1 M, pH 3.5, prepared using 0.1 M acetic acid solution and 0.1 M sodium acetate solution), and isoamylase (10 μL, 1400 U/mL) were added to the starch dispersion. The mixture was incubated in a water bath at 37 °C for 3 h. The debranched starch was precipitated using 5 mL of absolute ethanol, and centrifuged at 4000 r/min for 10 min, and finally, redissolved in 1 mL of DMSO/LiBr solution for 2 h at 80 °C in a thermomixer with shaking at 350 r/min.

The homogeneity and molecular weight of various fractions were measured using SEC-RI. The molecular weight distribution of debranched starch was analyzed using a differential refractive index detector (Optilab T-rEX, Wyatt Technology Co., Santa Barbara, CA, USA) equipped with two tandem columns (300 × 7.5 mm, Plael 10 μm MIXED-B and Plgel 5 μm MIXED-D; Agilent Technologies Inc, CA, USA) which was held at 80 °C using a model column heater. The flow rate is 0.8 mL/min. Standard dextrans of known molecular weights (342, 3650, 21000, 131,400, 610500, 821700, and 3755000 Da) were used for column calibration.

### 2.6 Attenuated total reflectance Fourier transform infrared (ATR-FTIR) spectroscopy

The infrared spectrometer (TenSor II 7800, Bruker Optics, Karlsruhe, Germany) was employed to record the FTIR spectra of the ternary complexes. The wavelength range of the scanning was 4000 to 400 cm^−1^, with a resolution of 4 cm^−1^ and a cumulative of 32 scans [24]. The spectral data were processed by OMNIC 9.2, and the spectra with wavelength range of 800–1200 cm^−1^ were specifically chosen for deconvolution analysis. The short-range ordered structure of complexes could be determined by the value of absorbance ratios at wavelengths of 1047 and 1022 cm^−1^ (R_1047/1022_).

### 2.7 Laser confocal micro-Raman (LCM-Raman) spectroscopy

Raman spectra of complexes were measured with a portable Raman spectrometer (BWS465-785 s, B&W, USA). The scanning range, integration time, laser power, and the resolution were 3200–400 cm^−1^, 10000 ms, 100 mW, and 4.5 cm^−1^ respectively. The full width at half-maximum (FWHM) of the characteristic absorption peak at 480 cm^−1^ was calculated by Wire 5.4 software to determine the short-range order of complexes [12].

### 2.8 X-ray diffraction (XRD)

The long-range order of the obtained ternary samples were determined by an X-ray diffractometer (Ultima IV, Rigaku Corporation, Kyoto, Japan) at 40 kV and 40 mA using Cu-Kα radiation(λ = 0.154 nm). The measurement involved scanning at a rate of 2°/min with a step size of 0.02° within the range of 5°-30° (2θ). The Jade 6.0 software was utilized to work out the ratio of V-peak area (around 13 and 20°) to total area, which was the relative crystallinity of the V-type complexes (Xv) [24].

### 2.9 Differential scanning calorimetry (DSC)

A differential scanning calorimetry (DSC214, Netzsch, Selbu, Germany) was used to measure the thermodynamic properties. Samples (3 mg) were placed at 40 μL DSC aluminum disc, followed by the addition of 9 μL of water. Then the crucible was sealed, and left to reach equilibrium at 25 °C throughout overnight. During the test, temperature rose from 20 °C to 120 °C at a steady rate of 10 °C/min, and the empty crucible was used as the reference. The thermodynamic parameters of the complex (melting onset temperature (T_o_), peak temperature (T_p_), conclusion temperature (T_c_) and enthalpy change (ΔH)) were recorded according to the DSC curves [13].

### 2.10 *In vitro* digestion

The *in vitro* enzymatic digestibility of the ternary complexes was assessed based on the method of Englyst et al [25] and Yan et al [26] with slightly modification. 2.6 g of porcine pancreatin was dissolved in 23.7 mL of distilled water, stirred at 37 °C for a duration of 10 min and then centrifuged at 3000 r/min for 20 min. Then 16 mL of supernatant was pipetted into a centrifuge tube and combined with 0.2 mL of amyloglucosidase to prepare the amylase mixture. 100 mg of starch-LA-βLG ternary complexes (dry basis) was mixed with 4 mL of sodium acetate buffer solution (0.1 mol/L, pH = 5.2). Those mixture was stabilized in water bath of 37 °C for 10 min, after which 1 mL of the mixed enzyme solution was added. Shock hydrolysis was carried out in a 37 °C water bath. After enzymolysis for 20 and 120 min, 0.1 mL of hydrolysate was extracted respectively, transferred into 4 mL of ethanol (70 %, v/v) immediately for inactivation, and then centrifuged for 10 min at 3000 r/min. The supernatant (0.1 mL) was extracted and blended with 3 mL of GOPOD under the protection from light conditions. Then the mixture was bathed in a 45℃ water for 20 min for chromogenic reaction.

0.1 mL of standard glucose solution was used as the standard, and distilled water was used as blank. The absorbance value was measured at a wavelength of 510 nm. The formula for calculating the contents of rapidly digestible starch (RDS), slowly digestible starch (SDS), and resistant starch (RS) was as follows [27]

$$\text{RDS}\left(\%\right)=\left({\text{G}}_{20}-\text{FG}\right)\times 0.9$$

$$\text{SDS}\left(\%\right)=\left({\text{G}}_{120}-{\text{G}}_{20}\right)\times 0.9$$

$$\text{RS}\left(\%\right)=1-\left(\text{RDS}+\text{SDS}\right)$$

where, G_20_ is the glucose content after enzymatic hydrolysis for 20 min, and G_120_ is the glucose content after enzymatic hydrolysis for 120 min. FG is the glucose content before enzymatic hydrolysis of the sample.

### 2.11 Statistical analysis

All of the experiments were repeated three times, and the data were expressed as value ± standard deviation (SD). Analysis of variance (ANOVA) by Duncan’s multiple range test (*p* < 0.05) were analyzed by SPSS 19.0 software (IBM Corporation, Armonk, NY, USA).

## 3 Results and discussion

### 3.1 Apparent amylose content

The amylose content of WS paste, MS paste, and PS paste after ultrasonic treatment were exhibited in Table 1. For WS, there was an increase in the amylose content from 16.26 % to 22.44 % as the ultrasonic power density was raised from 0 to 40 W/L. Ultrasonic treatment severed the α-1,6-glucosidic bonds of amylopectin, and broken down into some short amylopectin, which is treated as amylose [18], [28], thus causing the increasement of amylose content. For MS and PS, when the ultrasonic power density increased from 0 to 40 W/L, the amylose content increased from 17.32 % to 24.47 % and 20.98 % to 24.63 %, respectively. Nevertheless, at ultrasonic power density of 30 W/L, the amylose content slightly decreased, which may be due to the rearrangement of the dispersed amylose and the disintegration of amylose caused by ultrasound at 30 W/L [17].

**Table 1:** Amylose content and relative peak area of chain length distribution of ultrasonic pre-treated WS, MS, and PS.

Samples	AM content (%)	Peak area ratio (%)		
		Peak 1	Peak 2	Peak 3
WS-0	16.26 ± 0.31^e^	57.02 ± 0.15^c^	13.14 ± 0.09^b^	29.84 ± 0.05^a^
WS-10	17.97 ± 0.19^d^	57.52 ± 0.16^bc^	13.02 ± 0.05^b^	29.46 ± 0.11^b^
WS-20	19.88 ± 0.21^c^	57.81 ± 0.11^b^	13.11 ± 0.03^b^	29.08 ± 0.08^c^
WS-30	21.49 ± 0.22^b^	57.89 ± 0.06^b^	13.45 ± 0.07^a^	28.66 **±** 0.02^d^
WS-40	22.44 ± 0.17^a^	58.19 ± 0.03^a^	13.38 ± 0.08^a^	28.43 ± 0.11^e^
MS-0	17.32 ± 0.63^d^	58.90 ± 0.17^d^	15.21 ± 0.28^a^	25.89 ± 0.11^a^
MS-10	19.35 ± 0.25^c^	60.89 ± 0.18^c^	14.09 ± 0.12^bc^	25.02 ± 0.06^b^
MS-20	21.59 ± 0.09^b^	61.26 ± 0.06^b^	13.79 ± 0.07^d^	24.95 ± 0.13^b^
MS-30	19.64 ± 0.35^c^	61.33 ± 0.05^b^	14.03 ± 0.13^c^	24.64 ± 0.08^c^
MS-40	24.47 ± 0.56^a^	62.07 ± 0.03^a^	14.32 ± 0.09^b^	23.61 ± 0.06^d^
PS-0	20.98 ± 0.17^b^	48.78 ± 0.10^c^	29.82 ± 0.22^c^	21.40 ± 0.12^a^
PS-10	21.31 ± 0.21^b^	50.33 ± 0.09^b^	29.94 ± 0.08^c^	19.73 ± 0.17^b^
PS-20	24.09 ± 0.60^a^	50.60 ± 0.11^a^	29.82 ± 0.17^c^	19.58 ± 0.06^b^
PS-30	20.85 ± 0.35^b^	50.59 ± 0.05^a^	30.32 ± 0.11^b^	19.09 ± 0.16^c^
PS-40	24.63 ± 0.17^a^	50.27 ± 0.11^b^	30.86 ± 0.06^a^	18.87 ± 0.17^c^

Values are means ± SD. Values with the different letters for the same starch in the same column are significantly different (*p* < 0.05).

### 3.2 Chain length distribution (CLD)

**Fig. 1:**
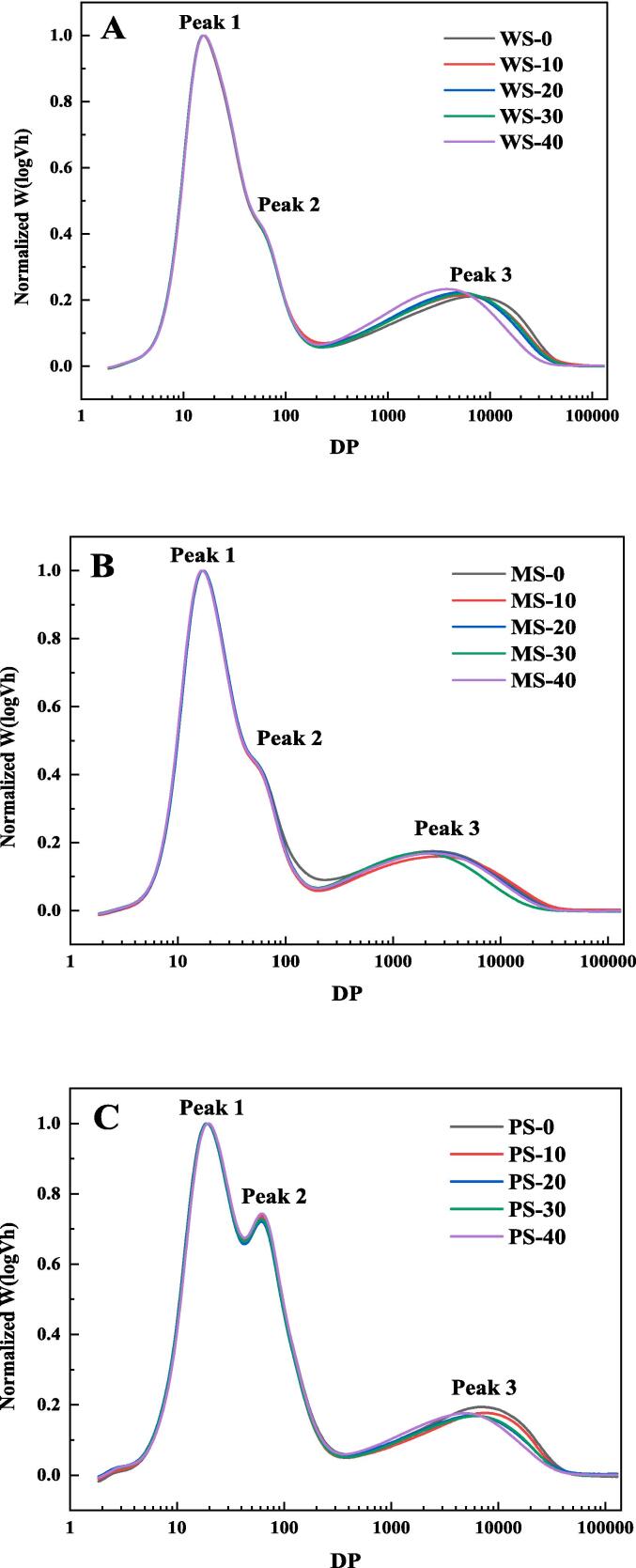
Chain length distribution of ultrasonic pre-treated WS (A), MS (B), and PS (C).

SEC-RI is a common method for the detection of molecular size and CLD. The degree of polymerization (DP), which was the number of glucose units in the molecular chain of starch, was used to measure the molecular size of starch. The CLD results of ultrasonic pre-treated WS, MS, and PS were shown in Fig. 1 and Table 1. There were three obvious peaks in Fig. 1. The first peak (denoted as peak 1) represented the shorter amylopectin chains confined to the single lamella with DP less than 30. The second peak (denoted as peak 2) represented longer amylopectin chains crossed two or more lamellae with DP ranging from 30 to 100. The third peak (denoted as peak 3) represented amylose chain with DP ranging from 100 to 20000 [29]. The area ratios of the three peaks changed after ultrasonic pretreatment for WS, MS and PS, indicating the variation of the length of starch chains. For WS, the area ratio of peak 3 decreased from 29.84 % to 28.43 % when the ultrasonic power density increased from 0 to 40 W/L, while the area ratios of peak 1 and peak 2 increased, indicating the shift of the chain length distribution towards the short chain direction due to the ultrasonic treatment [30]. For MS, the area ratio of peak 3 decreased from 25.89 % to 23.61 % when the ultrasonic power density increased from 0 to 40 W/L, meaning the decomposed of AM chains. The area ratio of peak 1 significantly increased, indicating the increased number of short chains with DP less than 30. The area ratio of peak 2 of MS-20 had the minimum value (13.79 %), meaning that the ultrasonic treatment at the power density of 20 W/L could shorten the longer amylopectin chains (DP ranging from 30 to 100). For PS, there was obvious reduction for PS-10 (19.73 %) compared with PS-0 (21.4 %), showing that PS was more sensitive to ultrasonic treatment, but the influence of ultrasonic power density variation was not obvious. Most of the amylose was broken down into short chains with DP less than 30 (peak 1), followed by chains with DP from 30 to 100 (peak 2). In short, ultrasonic treatment could reduce the content of chains with DP greater than 100 and increase the content of chains with DP less than 30.

### 3.3 Short-range ordered structure by ATR-FTIR

**Fig. 2:**
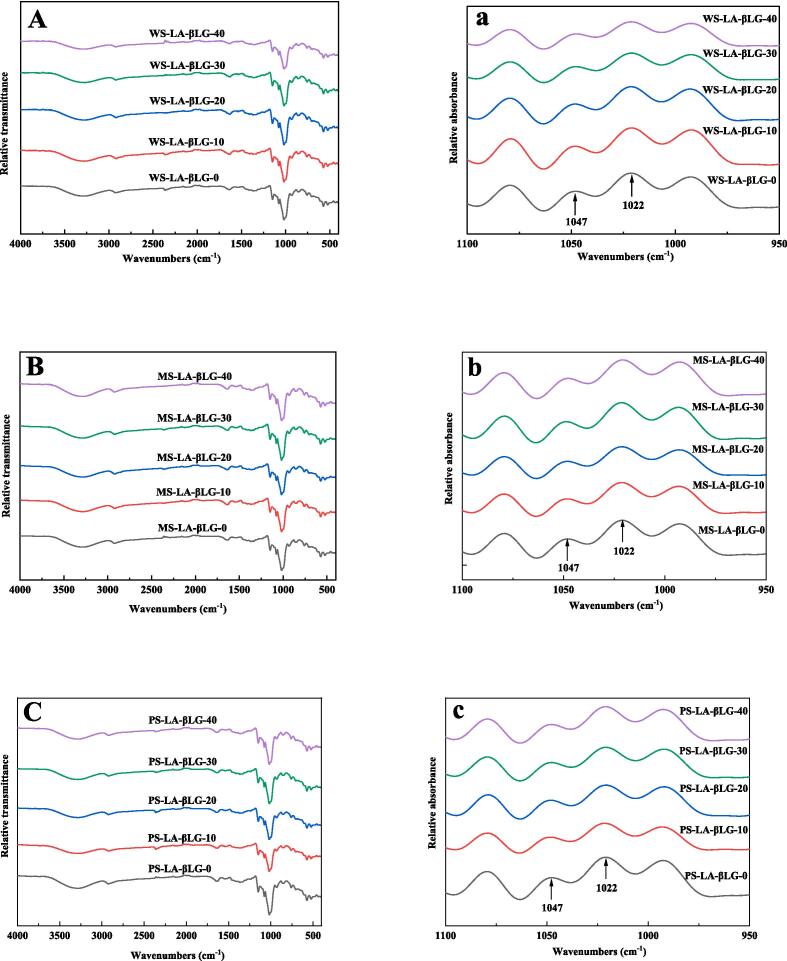
ATR-FTIR spectra of starch-LA-βLG ternary complexes with or without ultrasonic treatment. The original spectra: (A) WS-LA-βLG, (B) MS-LA-βLG, (C) PS-LA-βLG; The deconvoluted spectra: (a) WS-LA-βLG, (b) MS-LA-βLG, (c) PS-LA-βLG.

In FTIR spectra, the peaks at 1047 cm^−1^ and 1022 cm^−1^ were used to characterize the crystalline and amorphous structures of starch, respectively, and the absorbance ratio at 1047 and 1022 cm^−1^ (R_1047/1022_) was worded out to represent the short-range order of the complexes [31]. The higher R_1047/1022_, the greater short-range order exhibited by the complexes. According to Fig. 2 and Table 2, for the ternary complexes formed by WS, the R_1047/1022_ value increased from 0.567 to 0.637 as the ultrasonic power density rose from 0 to 10 W/L. However, as the ultrasonic power density continuously increased to 40 W/L, the R_1047/1022_ value decreased to 0.583. The highest R_1047/1022_ value at the ultrasonic power density of 10 W/L could be attributed to the formation of the largest amount of complexes [24], which resulted from the increasement of amylose content after ultrasonic pretreatment of starch. However, the inconsistency between the R_1047/1022_ value and amylose content when the ultrasonic power density was 20, 30, or 40 W/L might be attributed to the generated shorter amylose chains caused by excessive ultrasonic treatment (Table 1). The DP of amylose has been demonstrated to significantly affect the formation of amylose complexes, and the amylose with too short chains were hardly to form amylose complexes [5], [32]. The excessive ultrasonic treatment (20, 30, and 40 W/L) for WS could disrupt the starch structure by cleaving the starch chains into shorter chains, which became too brief to form WS-LA-βLG complexes, thereby reducing the value of R_1047/1022_. Similar results were displayed in previous researches [20], [33]. For MS and PS complexes, the R_1047/1022_ values increased as the ultrasonic power density increased from 0 to 20 W/L, and then decreased as the ultrasonic power density continually increased to 40 W/L. The highest R_1047/1022_ values for MS-LA-βLG-20 (0.615) and PS-LA-βLG-20 (0.630) meant that the most suitable ultrasonic power density for MS and PS to form ternary complexes was 20 W/L, which could be attributed to the augment of amylose content of gelatinized starch by ultrasonic treatment. Similarly, excessive ultrasonic treatment (30 and 40 W/L) could also inhibit the growth of MS-LA-βLG and PS-LA-βLG, which was consistent with WS-LA-βLG.

**Table 2:** R_1047/1022_, FWHM value and V-type relative crystallinity (Xv) of starch-LA-βLG ternary complexes with or without ultrasonic treatment.

Complexes	R_1047/1022_	FWHM (cm^−1^)	Xv (%)
WS-LA-βLG-0	0.567 ± 0.012^d^	16.86 ± 0.06^bc^	35.17 ± 0.44^b^
WS-LA-βLG-10	0.637 ± 0.011^a^	16.70 ± 0.04^c^	38.75 ± 0.05^a^
WS-LA-βLG-20	0.611 ± 0.009^b^	16.91 ± 0.18^bc^	35.73 ± 0.21^b^
WS-LA-βLG-30	0.607 ± 0.004^b^	17.00 ± 0.03^b^	33.09 ± 0.06^c^
WS-LA-βLG-40	0.583 ± 0.006^c^	17.30 ± 0.01^a^	32.75 ± 0.12^c^
MS-LA-βLG-0	0.563 ± 0.007^b^	17.52 ± 0.12^ab^	27.72 ± 0.09^d^
MS-LA-βLG-10	0.585 ± 0.025^ab^	17.44 ± 0.10^ab^	31.47 ± 0.07^c^
MS-LA-βLG-20	0.615 ± 0.025^a^	17.03 ± 0.25^b^	37.69 ± 0.11^a^
MS-LA-βLG-30	0.590 ± 0.024^ab^	17.23 ± 0.33^ab^	33.25 ± 0.32^b^
MS-LA-βLG-40	0.586 ± 0.007^ab^	17.60 ± 0.06^a^	31.58 ± 0.06^c^
PS-LA-βLG-0	0.553 ± 0.041^b^	17.50 ± 0.01^a^	31.08 ± 0.52^c^
PS-LA-βLG-10	0.603 ± 0.019^a^	17.05 ± 0.11^c^	34.57 ± 0.24^b^
PS-LA-βLG-20	0.630 ± 0.025^a^	16.76 ± 0.11^d^	37.44 ± 1.01^a^
PS-LA-βLG-30	0.607 ± 0.009^a^	17.17 ± 0.05^bc^	32.09 ± 0.61^c^
PS-LA-βLG-40	0.588 ± 0.003^ab^	17.36 ± 0.04^ab^	30.92 ± 0.48^c^

Values are means ± SD. Values with the different letters for the same starch complex in the same column are significantly different (*p* < 0.05).

### 3.4 Short-range ordered structure by LCM-Raman

In Raman spectrum, the FWHM of the characteristic peak at 480 cm^−1^ is often used to characterize the short-range order of starch [34]. The lower the FWHM value, the better the short-range order of the complexes [13]. It can be seen from Table 2 that WS-LA-βLG-10 showed the lowest FWHM value (16.7 cm^−1^), indicating the best short-range order when the WS-LA-βLG was formed at this condition [13]. This might be due to the formation of abundant WS-LA-βLG ternary complexes as stated in the analysis of the results of FTIR. For MS-LA-βLG and PS-LA-βLG, the lowest FWHM value was 17.03 cm^−1^ and 16.76 cm^−1^ respectively at the ultrasonic power density of 20 W/L, indicating the best short-range order of formed ternary complexes, which was consistent with the FTIR results. These results indicated that suitable ultrasonic treatment showed positive effects on the short-range order of ternary complexes.

### 3.5 Long-range ordered structure

**Fig. 3:**
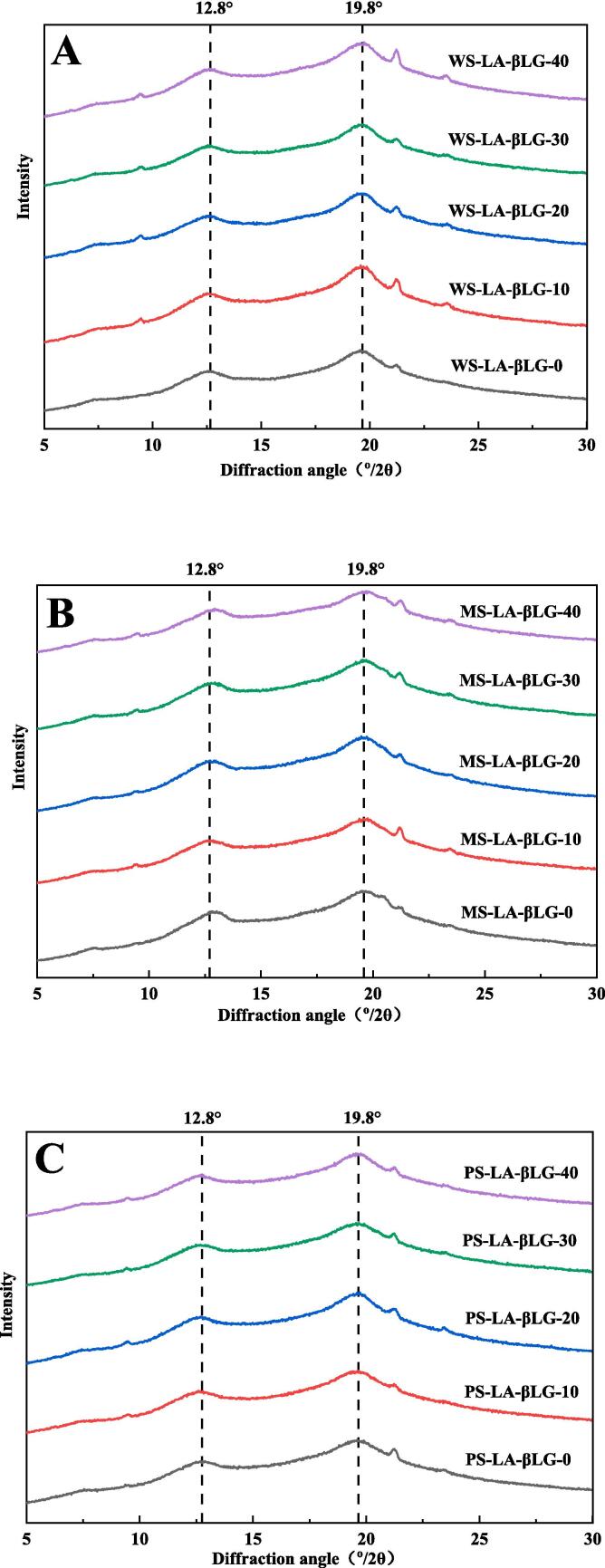
XRD patterns of starch-LA-βLG ternary complexes with or without ultrasonic treatment. (A) WS-LA-βLG (B) MS-LA-βLG (C) PS-LA-βLG.

The X-ray diffraction patterns (Fig. 3) and V-type relative crystallinity (Xv) (Table 2) of WS-LA-βLG, MS-LA-βLG, and PS-LA-βLG were used to characterize the long-range order of ternary complexes. The X-ray diffraction patterns showed that all samples had diffraction peaks at about 12.8° and 19.8°, indicating the generation of V-type starch-LA-βLG ternary complexes [35]. The other distinct peaks observed were ascribed to the uncomplexed LA [36]. In Fig. 3A, the intensity of peaks at about 12.8° and 19.8° for WS-LA-βLG-10 was stronger than that of other WS-LA-βLG complexes, indicating the best long-range order of WS-LA-βLG-10. Similar results can be exhibited for MS-LA-βLG-20 and PS-LA-βLG-20 in Fig. 3B and 3C, which had greater intensity of peaks at about 12.8° and 19.8° than other corresponding ternary complexes.

The Xv values of WS-LA-βLG after ultrasonic treatment at different powers density initially increased and then decreased as ultrasonic power density increased. The maximum value of Xv (38.75 %) was obtained at ultrasonic power density of 10 W/L, indicating the best long-range order of WS-LA-βLG-10, which might be owing to the formation of more ternary complexes [12], [13]. With the further increase of ultrasonic power density, the Xv value decreased to 32.75 % at the ultrasonic power density of 40 W/L. For MS-LA-βLG and PS-LA-βLG, the tendency was similar to that of WS-LA-βLG, while the highest value of Xv displayed at the ultrasonic power density of 20 W/L, which was 37.69 % and 37.44 %, respectively. With the ultrasonic power density increasing to 40 W/L, it gradually decreased to 31.58 % and 30.92 %, respectively. These results indicated that low degree of ultrasonic treatment could improve the long-range order of the ternary complexes, while excessive ultrasonic treatment may inhibit the formation of ordered structure. What’s more, the optimal ultrasonic power density for different types of starches was different.

### 3.6 Thermal properties

**Fig. 4:**
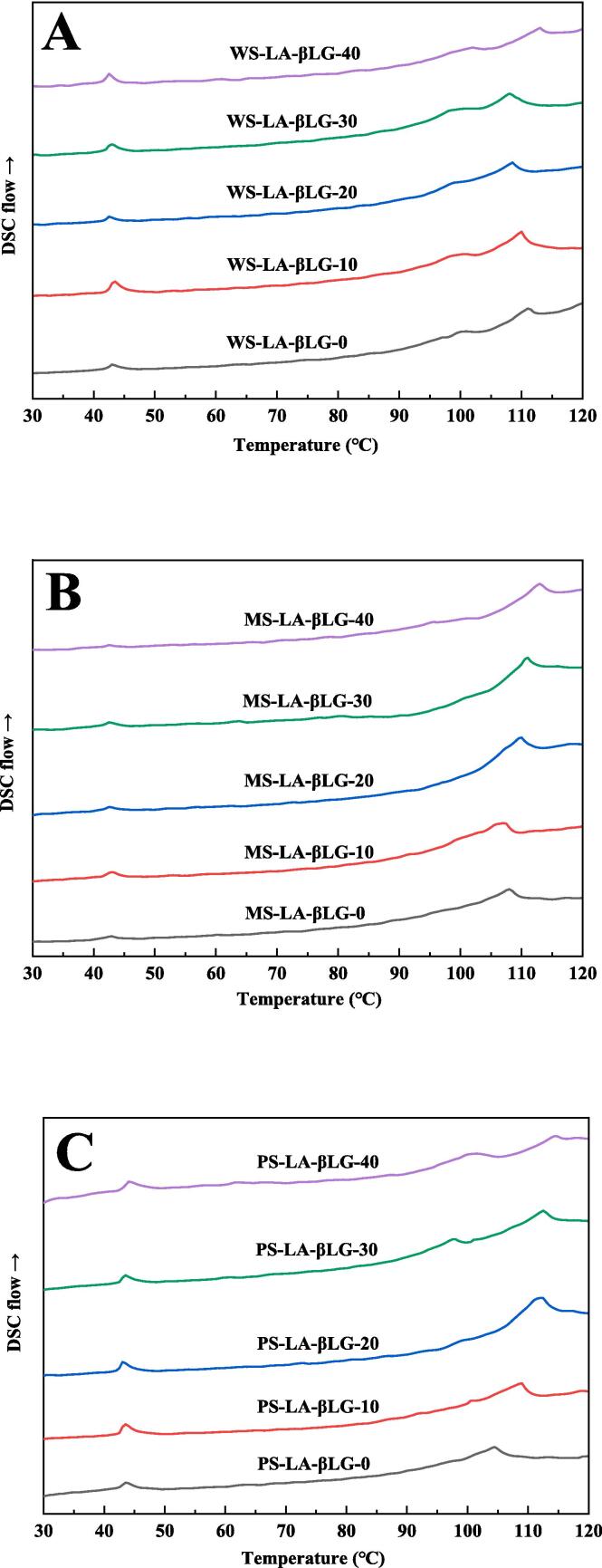
DSC curves of starch-LA-βLG ternary complexes with or without ultrasonic treatment. (A) WS-LA-βLG (B) MS-LA-βLG (C) PS-LA-βLG.

The DSC curves (Fig. 4) and thermodynamic parameters (Table 3) were obtained to measure the thermal properties of WS-LA-βLG, MS-LA-βLG and PS-LA-βLG. The information on the stability of the structure was provided by the temperatures of endothermic transitions, and the amounts of ordered structure was reflected by the change in enthalpy [4]. In Fig. 4, the endothermic peaks at around 43 °C were ascribed to the melting of lauric acid which was not included in the complexes [37]. For WS-LA-βLG, there were two endothermic transitions at around 100 and 110 °C, which were thought to be caused by the melting of type Ⅰ and type Ⅱ ternary complexes, respectively [37]. Differently, only one endothermic transition was presented for MS-LA-βLG. However, it is interesting to note that the endothermic transition changed into two peaks for PS-LA-βLG when the ultrasonic power density was increased to 20, 30, and 40 W/L, which can also be seen from Table 3. These results demonstrated that ultrasonic treatment with higher power density could alter the crystallization type of the PS-LA-βLG complexes. Moreover, the Tp value for the two types PS complexes rose with the increase of ultrasonic power density, indicating that more stable crystalline structure was formed in PS-LA-βLG complexes at higher ultrasonic power density.

**Table 3:** Thermal properties of starch-LA-βLG ternary complexes with or without ultrasonic treatment.

Complexes	To_Ⅰ_(℃)	Tp_Ⅰ_(℃)	Tc_Ⅰ_(℃)	ΔH_Ⅰ_(J/g)	To_Ⅱ_(℃)	Tp_Ⅱ_(℃)	Tc_Ⅱ_(℃)	ΔH_Ⅱ_ (J/g)	ΔH_T_ (J/g)
WS-LA-βLG-0	99.70 ± 1.70^ab^	100.65 ± 0.49^a^	103.10 ± 0.57^a^	1.36 ± 0.05^b^	107.25 ± 0.35^a^	110.55 ± 1.06^bc^	112.85 ± 1.06^b^	2.67 ± 0.31^c^	4.04 ± 0.36^b^
WS-LA-βLG-10	95.55 ± 0.07^c^	101.00 ± 0.42^a^	102.95 ± 0.49^a^	1.47 ± 0.16^ab^	105.95 ± 0.49^ab^	109.70 ± 0.42^c^	114.80 ± 0.71^a^	5.71 ± 0.04^a^	7.18 ± 0.19^a^
WS-LA-βLG-20	97.30 ± 0.57^bc^	99.30 ± 0.71^a^	102.45 ± 0.21^a^	0.69 ± 0.02^c^	105.65 ± 0.49^ab^	108.25 ± 0.35^d^	112.40 ± 0.57^b^	3.43 ± 0.40^b^	4.11 ± 0.43^b^
WS-LA-βLG-30	100.15 ± 1.48^a^	101.45 ± 0.35^a^	103.75 ± 0.49^a^	0.69 ± 0.05^c^	107.15 ± 0.78^a^	111.20 ± 0.00^b^	112.40 ± 0.42^b^	3.03 ± 0.01^bc^	3.72 ± 0.04^b^
WS-LA-βLG-40	95.85 ± 0.35^c^	99.10 ± 4.95^a^	102.90 ± 1.13^a^	1.57 ± 0.00^a^	104.85 ± 0.07^b^	112.80 ± 0.28^a^	114.00 ± 0.14^ab^	2.08 ± 0.01^d^	3.66 ± 0.01^b^
MS-LA-βLG-0					104.30 ± 0.14^b^	107.90 ± 0.00^ab^	109.45 ± 0.07^b^	4.65 ± 0.39^c^	4.65 ± 0.39^c^
MS-LA-βLG-10					101.20 ± 0.14^c^	107.00 ± 0.28^b^	108.55 ± 0.07^b^	5.80 ± 0.64^b^	5.80 ± 0.64^b^
MS-LA-βLG-20					100.87 ± 0.12^c^	107.17 ± 3.65^b^	108.73 ± 3.21^b^	7.83 ± 0.06^a^	7.83 ± 0.06^a^
MS-LA-βLG-30					103.80 ± 0.92^b^	109.25 ± 0.78^ab^	111.10 ± 0.57^ab^	6.92 ± 0.40^a^	6.92 ± 0.40^a^
MS-LA-βLG-40					108.85 ± 0.35^a^	112.37 ± 1.18^a^	113.70 ± 0.89^a^	5.86 ± 0.62^b^	5.86 ± 0.62^b^
PS-LA-βLG-0					94.03 ± 0.31^d^	104.77 ± 0.31^c^	106.40 ± 0.17^c^	4.64 ± 0.20^c^	4.64 ± 0.20^c^
PS-LA-βLG-10					99.43 ± 0.49^c^	107.90 ± 0.10^b^	109.57 ± 0.15^b^	5.32 ± 0.11^b^	5.32 ± 0.11^b^
PS-LA-βLG-20	96.70 ± 0.28^ab^	98.55 ± 0.21^a^	100.95 ± 0.35^b^	0.30 ± 0.04^c^	107.05 ± 0.49^b^	113.10 ± 1.13^a^	114.65 ± 0.78^a^	7.52 ± 0.17^a^	7.82 ± 0.13^a^
PS-LA-βLG-30	98.35 ± 1.63^a^	99.70 ± 2.83^a^	101.90 ± 1.27^b^	1.38 ± 0.36^b^	107.15 ± 1.48^b^	112.90 ± 0.42^a^	114.30 ± 0.42^a^	3.82 ± 0.04^d^	5.20 ± 0.40^b^
PS-LA-βLG-40	94.65 ± 0.49^b^	102.15 ± 0.64^a^	104.55 ± 0.49^a^	3.22 ± 0.29^a^	109.20 ± 1.41^a^	113.35 ± 1.63^a^	114.55 ± 1.77^a^	1.60 ± 0.09^e^	4.82 ± 0.21^bc^

Values are means ± SD. Values with the different letters for the same starch complex in the same column are significantly different (*p* < 0.05).

The experimental results showed that the total enthalpy change (ΔH_T_) values increased first and subsequently decreased as the ultrasonic power density increased. When the ultrasonic power density was 10 W/L, the ΔH_T_ of WS-LA-βLG reached the maximum of 7.18 J/g. ΔH_T_ values exhibited a positive correlation with the amount of complexes [4]. Ultrasonic treatment increased the AM content, so the content of the complex increased accordingly [38]. The subsequent reduction of ΔH_T_ values was due to the too short amylose chains caused by excessive ultrasonic treatment, which were hardly to form amylose complexes. The ΔH_T_ values of MS-LA-βLG and PS-LA-βLG also initially increased and subsequently decreased with the increment of ultrasonic power density, and the highest value was 7.83 J/g and 7.82 J/g respectively when the selected ultrasonic power density reached to 20 W/L. In a word, ultrasonic treatment could facilitate the formation of complexes and improve their thermostability at appropriate ultrasonic power density. This was consistent with XRD, FTIR, and Raman results.

### 3.7 *In vitro* digestion properties

**Fig. 5:**
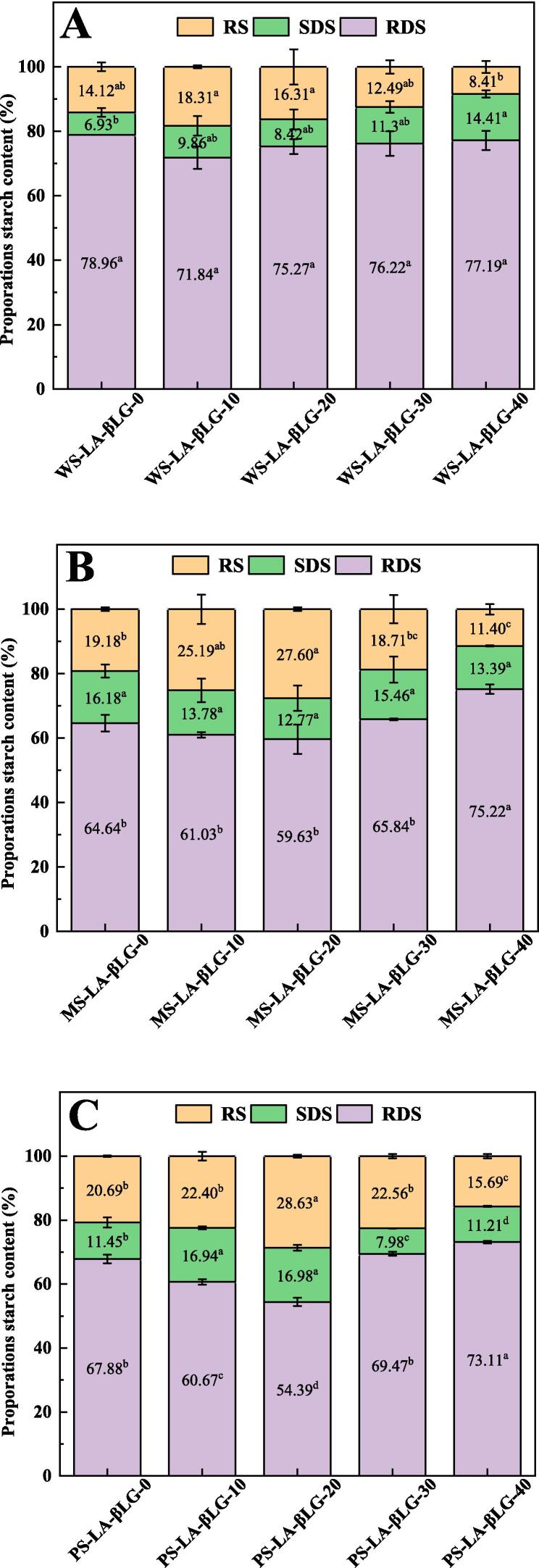
RDS, SDS, and RS content of starch-LA-βLG ternary complexes with or without ultrasonic treatment. (A) WS-LA-βLG (B) MS-LA-βLG (C) PS-LA-βLG.

The contents of RDS, SDS, and RS of WS-LA-βLG, MS-LA-βLG and PS-LA-βLG complexes under diverse ultrasonic treatments were exhibited in Fig. 5. The RS content of WS-LA-βLG complexes rose from 14.12 % to 18.31 % as the ultrasonic power density reached to 10 W/L, and then decreased to 8.4 % with the ultrasonic power density enhanced to 40 W/L. An opposite tendency was observed for RDS contents. These findings were in agreement with the variations observed in the amount of WS-LA-βLG complexes. Starch-lipid-protein complexes have been proved to reduce the digestibility of starch due to a more compact crystal structure and enhanced structural order, which heightened steric hindrance and reduced the binding sites of starch to the enzyme [4], [39]. Similar results were found for MS and PS complexes. The RS content of MS-LA-βLG and PS-LA-βLG complexes increased from 19.18 % and 20.69 % to 27.60 % and 28.63 % respectively when the ultrasonic power density increased to 20 W/L. Then the RS content decreased with the ultrasonic power density continuously increased to 30 and 40 W/L. The RDS content was lowest at the ultrasonic power density of 20 W/L for MS-LA-βLG and PS-LA-βLG complexes. The findings on digestibility suggested that a certain ultrasonic power density was benefit for the formation of the complexes, and then increase the content of RS, which has potential applications in foods with low GI.

## 4 Conclusion

This study investigated the effects of starch (WS, MS, and PS) with different degree of ultrasonic treatment on the formation of starch-LA-βLG complexes. Ultrasonic treatment increased the amylose content in all three types of starch and shifted their chain length distribution towards the short chains. FTIR, Raman, and XRD results showed that the best short- and long- range order structure for WS-LA-βLG was achieved at 10 W/L, while for MS-LA-βLG and PS-LA-βLG, it was at 20 W/L. DSC results showed that WS formed the largest amount of complexes at 10 W/L, and MS and PS did so at 20 W/L. Additionally, higher ultrasonic power density altered the PS complexes into two crystal forms (Ⅰ and Ⅱ). In addition, ultrasound could also improve the RS content of the complex, suggesting its potential use in preparing low GI food. However, this research was conducted using a model system of starch, lipids, and proteins, and the impact of ultrasonic treatment on real starch-based food is not yet known. Therefore, enhancing the interactions of components in starch-based food through ultrasonic treatment to improve their quality will be an important field of future research.

## CRediT authorship contribution statement

**Bin Niu:** Writing – original draft, Funding acquisition, Conceptualization. **Yingnan Qin:** Methodology, Data curation. **Xinhua Xie:** Supervision, Formal analysis. **Bobo Zhang:** Formal analysis. **Lilin Cheng:** Formal analysis. **Yizhe Yan:** Writing – review & editing, Funding acquisition.

## Declaration of competing interest

The authors declare that they have no known competing financial interests or personal relationships that could have appeared to influence the work reported in this paper.

## References

[1] Liu X., Huang S., Chao C., Yu J., Copeland L., Wang S. (2022). Changes of starch during thermal processing of foods: Current status and future directions. Trends Food Sci. Tech..

[2] Masina N., Choonara Y.E., Kumar P., du Toit L.C., Govender M., Indermun S., Pillay V. (2017). A review of the chemical modification techniques of starch. Carbohyd. Polym..

[3] Obiro W.C., Sinha Ray S., Emmambux M.N. (2012). V-amylose Structural Characteristics, Methods of Preparation, Significance, and Potential Applications. Food Rev. Int..

[4] Putseys J.A., Lamberts L., Delcour J.A. (2010). Amylose-inclusion complexes: Formation, identity and physico-chemical properties. J. Cereal Sci..

[5] Wang S., Chao C., Cai J., Niu B., Copeland L., Wang S. (2020). Starch-lipid and starch-lipid-protein complexes: A comprehensive review. Compr. Rev. Food Sci. f..

[6] Duan Y., Chao C., Yu J., Liu Y., Wang S. (2023). Effects of different sources of proteins on the formation of starch-lipid-protein complexes. Int J Biol Macromol..

[7] Zheng M., Chao C., Yu J., Copeland L., Wang S., Wang S. (2018). Effects of Chain Length and Degree of Unsaturation of Fatty Acids on Structure and in Vitro Digestibility of Starch-Protein-Fatty Acid Complexes. J. Agric. Food Chem..

[8] Chen W., Chao C., Yu J., Copeland L., Wang S., Wang S. (2021). Effect of protein-fatty acid interactions on the formation of starch-lipid-protein complexes. Food Chem..

[9] Zhang G., Maladen M., Campanella O.H., Hamaker B.R. (2010). Free fatty acids electronically bridge the self-assembly of a three-component nanocomplex consisting of amylose, protein, and free fatty acids. J. Agric. Food Chem..

[10] Wang C., Chao C., Yu J., Copeland L., Huang Y., Wang S. (2022). Mechanisms Underlying the Formation of Amylose-Lauric Acid-β-Lactoglobulin Complexes: Experimental and Molecular Dynamics Studies. J. Agric. Food Chem..

[11] Gutiérrez T.J., Tovar J. (2021). Update of the concept of type 5 resistant starch (RS5): Self-assembled starch V-type complexes. Trends Food Sci. Tech..

[12] Niu B., Chao C., Cai J., Yan Y., Copeland L., Yu J., Wang S., Wang S. (2020). Effect of pH on formation of starch complexes with lauric acid and β-lactoglobulin. LWT-Food Sci. Technol..

[13] Niu B., Chao C., Cai J., Yu J., Wang S., Wang S. (2021). Effects of cooling rate and complexing temperature on the formation of starch-lauric acid-β-lactoglobulin complexes. Carbohyd. Polym..

[14] Zhou C., Okonkwo C.E., Inyinbor A.A., Yagoub A.E.A., Olaniran A.F. (2023). Ultrasound, infrared and its assisted technology, a promising tool in physical food processing: A review of recent developments. Crit. Rev. Food Sci. Nutr..

[15] Bonto A.P., Tiozon R.N., Sreenivasulu N., Camacho D.H. (2021). Impact of ultrasonic treatment on rice starch and grain functional properties: A review. Ultrason. Sonochem..

[16] Gaquere-Parker A., Taylor T., Hutson R., Rizzo A., Folds A., Crittenden S., Zahoor N., Hussein B., Arruda A. (2018). Low frequency ultrasonic-assisted hydrolysis of starch in the presence of α-amylase. Ultrason. Sonochem..

[17] Xiao Y., Wu X., Zhang B., Luo F., Lin Q., Ding Y. (2021). Understanding the aggregation structure, digestive and rheological properties of corn, potato, and pea starches modified by ultrasonic frequency. Int. J. Biol. Macromol..

[18] Zhang B., Xiao Y., Wu X., Luo F., Lin Q., Ding Y. (2021). Changes in structural, digestive, and rheological properties of corn, potato, and pea starches as influenced by different ultrasonic treatments. Int. J. Biol. Macromol..

[19] Kang X., Liu P., Gao W., Wu Z., Yu B., Wang R., Cui B., Qiu L., Sun C. (2020). Preparation of starch-lipid complex by ultrasonication and its film forming capacity. Food Hydrocolloid..

[20] Zhang X., Mi T., Gao W., Wu Z., Yuan C., Cui B., Dai Y., Liu P. (2022). Ultrasonication effects on physicochemical properties of starch-lipid complex. Food Chem..

[21] Zhuang J., Liu H., You L., Xu F., Zeng H., Zeng S. (2023). Influence of ultrasonic-microwave power on the structure and in vitro digestibility of lotus seed starch-glycerin monostearin complexes after retrogradation. Int. J. Biol. Macromol..

[22] Chumsri P., Panpipat W., Cheong L.-Z., Chaijan M. (2022). Formation of Intermediate Amylose Rice Starch-Lipid Complex Assisted by Ultrasonication. Foods..

[23] Li Y., Hu A., Zheng J., Wang X. (2019). Comparative studies on structure and physiochemical changes of millet starch under microwave and ultrasound at the same power. Int. J. Biol. Macromol..

[24] Niu B., Qin Y., Zhu X., Zhang B., Cheng L., Yan Y. (2024). Effect of plasma-activated water on the formation of endogenous wheat starch-lipid complexes during extrusion. Int. J. Biol. Macromol..

[25] Englyst H.N., Kingman S.M., Cummings J.H. (1992). Classification and measurement of nutritionally important starch fractions. Eur. J. Clin. Nutr..

[26] Yan Y., Feng L., Shi M., Cui C., Liu Y. (2020). Effect of plasma-activated water on the structure and in vitro digestibility of waxy and normal maize starches during heat-moisture treatment. Food Chem..

[27] Yan Y., An H., Liu Y., Ji X., Shi M., Niu B. (2023). Debranching facilitates malate esterification of waxy maize starch and decreases the digestibility. Int. J. Biol. Macromol..

[28] Liu P., Kang X., Cui B., Gao W., Wu Z., Yu B. (2019). Effects of amylose content and enzymatic debranching on the properties of maize starch-glycerol monolaurate complexes. Carbohyd. Polym..

[29] Xing B., Yang X., Zou L., Liu J., Liang Y., Li M., Zhang Z., Wang N., Ren G., Zhang L., Qin P. (2023). Starch chain-length distributions determine cooked foxtail millet texture and starch physicochemical properties. Carbohyd. Polym..

[30] Han L., Huang J., Yu Y., Thakur K., Wei Z., Xiao L., Yang X. (2023). The alterations in granule, shell, blocklets, and molecular structure of pea starch induced by ultrasound. Int. J. Biol. Macromol..

[31] Ashwar B.A., Gani A., Wani I.A., Shah A., Masoodi F.A., Saxena D.C. (2016). Production of resistant starch from rice by dual autoclaving-retrogradation treatment: Invitro digestibility, thermal and structural characterization. Food Hydrocolloid..

[32] Gelders G.G., Vanderstukken T.C., Goesaert H., Delcour J.A. (2004). Amylose–lipid complexation: a new fractionation method. Carbohyd. Polym..

[33] Li J., Tian L., Fang Y., Chen W., Huang G. (2020). Ultrasonic-Assisted Preparation of Maize Starch-Caffeic Acid Complex: Physicochemical and Digestion Properties. Starch-Stärke.

[34] Zhou Y., Yan Y., Shi M., Liu Y. (2018). Effect of an Atmospheric Pressure Plasma Jet on the Structure and Physicochemical Properties of Waxy and Normal Maize Starch. Polymers.

[35] Li X., Wang C., Chao C., Yu J., Copeland L., Liu Y., Wang S. (2023). Prior interaction of protein and lipid affects the formation of ternary complexes with starch. Food Chem..

[36] Niu B., Chao C., Cai J., Yan Y., Copeland L., Wang S., Wang S. (2019). The effect of NaCl on the formation of starch-lipid complexes. Food Chem..

[37] Wang J., Yu J., Copeland L., Wang S. (2023). Revisiting the Formation of Starch-Monoglyceride-Protein Complexes: Effects of Octenyl Succinic Anhydride Modification. J. Agric. Food Chem..

[38] He H., Zheng B., Wang H., Li X., Chen L. (2020). Insights into the multi-scale structure and in vitro digestibility changes of rice starch-oleic acid/linoleic acid complex induced by heat-moisture treatment. Food Res. Int..

[39] Kang X., Jia S., Gao W., Wang B., Zhang X., Tian Y., Sun Q., Atef M., Cui B., Abd El-Aty A.M. (2022). The formation of starch-lipid complexes by microwave heating. Food Chem..

